# Prematurity is associated with white matter T2 MRI visible perivascular spaces in very preterm-born neonates

**DOI:** 10.1093/braincomms/fcaf244

**Published:** 2025-06-18

**Authors:** Lena Meinhold, Antonio G Gennari, Giancarlo Natalucci, Beatrice Latal, Flavia M Wehrle, Jean-Claude Fauchere, Cornelia Hagmann, Ruth O'Gorman Tuura

**Affiliations:** Center for MR Research, University Children’s Hospital Zurich, 8008 Zurich, Switzerland; Children’s Research Centre, University Children’s Hospital Zurich, 8008 Zurich, Switzerland; University of Zurich, Medical Faculty, 8032 Zurich, Switzerland; Center for MR Research, University Children’s Hospital Zurich, 8008 Zurich, Switzerland; Children’s Research Centre, University Children’s Hospital Zurich, 8008 Zurich, Switzerland; University of Zurich, Medical Faculty, 8032 Zurich, Switzerland; Department of Neonatology, University Hospital Zurich, 8091 Zurich, Switzerland; Children’s Research Centre, University Children’s Hospital Zurich, 8008 Zurich, Switzerland; University of Zurich, Medical Faculty, 8032 Zurich, Switzerland; Child Development Centre, University Children’s Hospital Zurich, 8008 Zurich, Switzerland; Children’s Research Centre, University Children’s Hospital Zurich, 8008 Zurich, Switzerland; University of Zurich, Medical Faculty, 8032 Zurich, Switzerland; Child Development Centre, University Children’s Hospital Zurich, 8008 Zurich, Switzerland; Department of Neonatology, University Hospital Zurich, 8091 Zurich, Switzerland; Children’s Research Centre, University Children’s Hospital Zurich, 8008 Zurich, Switzerland; University of Zurich, Medical Faculty, 8032 Zurich, Switzerland; Department of Neonatology, University Children’s Hospital Zurich, 8008 Zurich, Switzerland; Center for MR Research, University Children’s Hospital Zurich, 8008 Zurich, Switzerland; Children’s Research Centre, University Children’s Hospital Zurich, 8008 Zurich, Switzerland; University of Zurich, Medical Faculty, 8032 Zurich, Switzerland

**Keywords:** very preterm birth, perivascular spaces, glymphatic pathway, magnetic resonance imaging, neurofluids

## Abstract

Perivascular spaces (PVSs), as biomarkers of the brain’s neurofluid clearance system, remain largely unexplored in the brains of very preterm and term-born infants. In this retrospective cross-sectional study, we investigate PVSs in very preterm and term-born neonates and explore potential associations with preterm birth, maturation, brain injury and developmental outcome. T2-weighted fast spin echo MRI data of 86 very preterm (<32 gestational weeks) and 43 term-born neonates were acquired at term-equivalent age (mean postmenstrual age = 41.63 ± 2.09 weeks) with a 3T GE HD.xt scanner, using an 8-channel head coil, with echo time/repetition time = 109/5700 ms, field of view = 25.6 cm, acquisition matrix = 256 × 256, reconstruction matrix = 512 × 512 and slice thickness = 2 mm. PVS counts were estimated visually in the basal ganglia and centrum semiovale (CSO) using a validated scoring system. Developmental outcome was evaluated using the Bayley Scales of Infant Development II or III. White and grey matter injuries were evaluated using Woodward’s score. Groupwise differences in PVS counts between term-born and very preterm-born neonates and associations with postmenstrual age, preterm birth, brain injury and developmental outcomes were evaluated with Mann–Whitney tests and multiple regression. PVS counts in the basal ganglia did not differ between groups, whereas PVS counts in the CSO were significantly higher in very preterm-born neonates (median = 1, interquartile range: 0–2), compared with term-born neonates (median = 0, interquartile range: 0–0; *P* < 0.001). CSO PVSs were independently associated with postmenstrual age, incidence rate ratio = 1.44, confidence interval 1.23–1.70), *P* < 0.001, and preterm birth, incidence rate ratio = 44.87, confidence interval 10.39–212.98, *P* < 0.001. Including a postmenstrual age-by-group interaction showed that the maturation-related increase of CSO PVSs did not differ between the groups. We found no evidence for an association of PVSs with brain injury (grey matter injury: *P* = 0.75 for basal ganglia and *P* = 0.84 for CSO; white matter injury: *P* = 0.92 for basal ganglia and *P* = 0.60 for CSO) or developmental outcome (*P* = 0.31 for basal ganglia, *P* = 0.24 for CSO). These results demonstrate an association of CSO PVSs with preterm birth and an increase of CSO PVSs with maturation. Longitudinal studies may shed further light on the role of preterm birth on the brain’s neurofluid clearance system development.

## Teaser

Meinhold *et al*. investigated MRI-visible perivascular spaces in neonates and report that white matter perivascular spaces are increased in very preterm infants and associated with both maturation and preterm birth. No associations were found with brain injury or developmental outcomes.

## Graphical Abstract

**Graphical Abstract:**
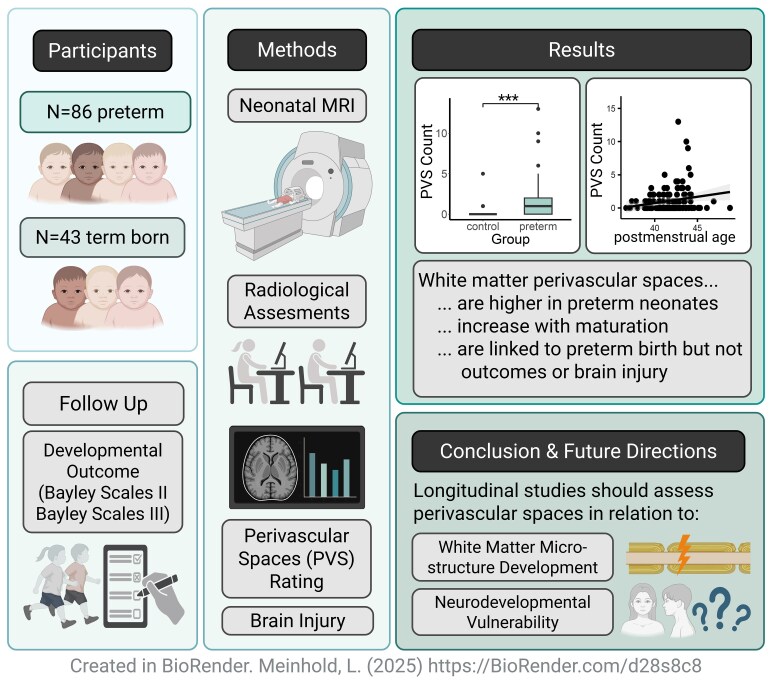

## Introduction

### Perivascular spaces and the brain’s neurofluid system

Perivascular spaces (PVSs) are fluid-filled spaces surrounding small, cerebral, penetrating blood vessels. They form part of the brain's neurofluid system, through which they are involved in brain fluid and ion homeostasis, immune^1^ and waste clearance functions via the recently posited ‘glymphatic’ pathway.^2^ It is known that PVSs may become enlarged with ageing,^3^ during inflammation^4-6^ or with vascular risk factors.^7^ These morphological changes are visually observable on T2-weighted MRI images and are thought to reflect underlying disruptions to glymphatic function.^8^ Consequently, enlarged PVSs have been identified in several central nervous system disorders and have been suggested as biomarkers reflecting the functioning of the brain’s neurofluid system.^8-11^

### Perivascular spaces across development

Data from large cross-sectional studies suggest that PVS counts, volume and diameter in healthy individuals undergo dynamic, physiological changes across the lifespan.^12,13^ For white matter PVSs, the most rapid increases happen early in life, whereas basal ganglia (BG) PVSs are thought to decrease in childhood and increase again in adulthood.^12,14^ These studies report data from individuals aged 8 years or older; therefore, up until now, PVSs have been barely studied in the brains of younger children and neonates. Only one study investigated PVSs in preterm and term-born neonates, demonstrating that the BG PVS fraction is inversely related to maturation and that preterm-born neonates had smaller BG PVS volumes compared with term-born neonates.^14^

### The developing neurofluid system

Although the development of the neurofluid system in early life has not been directly examined in humans, studies investigating its inherent structures and functions point towards an incomplete state of maturation, both structurally and functionally, in neonates and even more so in preterms. For example, preterm-born neonates show incomplete cerebral vascularization and autoregulation,^15^ and in contrast to the body’s lymphatic system, meningeal lymphatic vessels have been shown to form in the postnatal period.^16-18^ Moreover, astrocytes, which associate with perivascular structures through their endfeet, continue to mature postnatally and become fully mature by postnatal Week 3 or 4 in humans.^19,20^ Therefore, preterm birth may overlap with a critical window in the development of the neurofluid system and potentially interfere with its healthy maturation, making PVSs an important target of investigation in this population.

### Causes and consequences of preterm birth and their potential effects on the perivascular unit

Preterm birth is defined as any birth happening before 37 completed weeks of gestation, and births occurring between 28 and 32 weeks are considered very preterm.^21^ Preterm birth is commonly categorized as either spontaneous or iatrogenic. Spontaneous preterm birth accounts for the majority of cases and includes preterm labour with or without preterm prelabour rupture of membranes, often associated with maternal or foetal infection or inflammation. Iatrogenic preterm birth, on the other hand, results from medically indicated early delivery due to maternal or foetal complications.^22,23^ Consequences of preterm birth in the postnatal period include hypoxic-ischaemic events, intraventricular haemorrhage (IVH) and white matter injury.^24-26^ Inflammation caused by infection may already be present in the foetus before birth or may arise after birth as a consequence of hypoxia-ischaemia.^15^ These processes may affect perivascular structures and may, for example, lead to perivascular enlargement in response to immune activation^4-6,27^ which may be visible on T2-weighted MRI images after birth. Furthermore, prematurity may lead to unfavourable neurodevelopmental outcomes, including cognitive, motor, or language impairments.^24,28,29^ It is currently unclear whether insults on the maturing perivascular unit resulting from prematurity could also affect these outcomes, e.g. via disturbed metabolite clearance or ion homeostasis.^30^ Thus, the purpose of this study was to assess PVS counts in very preterm and term-born neonates and explore potential associations of PVSs with preterm birth, maturation, white and grey matter injuries and developmental outcome.

## Materials and methods

### Study sample

**Figure 1:**
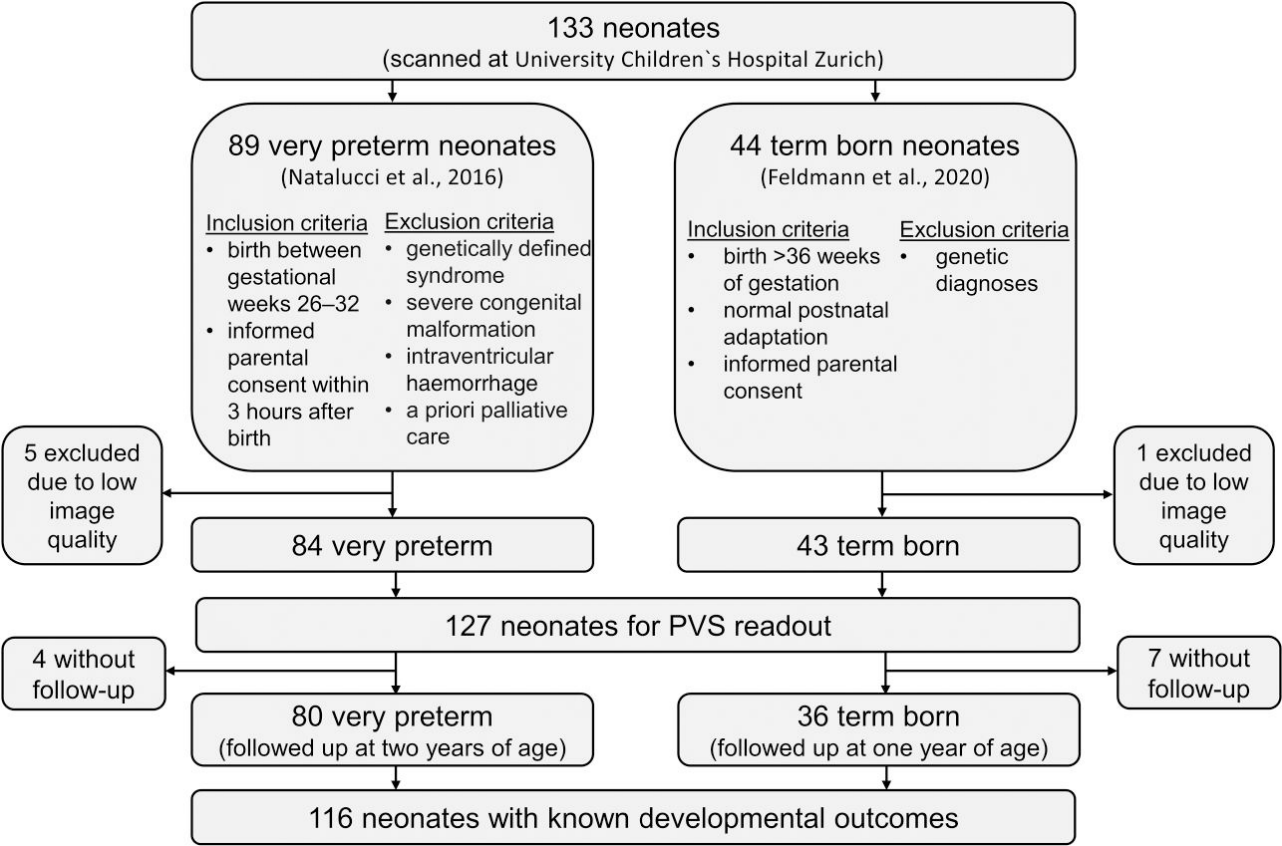
Sample inclusion and exclusion criteria flow chart.

In this retrospective cross-sectional study, we included 89 very preterm-born neonates who were initially enrolled in a randomized controlled trial, investigating the effect of early high-dose recombinant human erythropoietin (rhEPO) on neurodevelopmental outcome at 2 years (clinicaltrials.gov identifier: NCT00413946).^31^ The multicentre Phase 3 clinical trial took place in Switzerland between 2005 and 2012. Inclusion criteria were birth between gestational Weeks 26 and 32 and informed parental consent within 3 h after birth. Exclusion criteria were a genetically defined syndrome, a severe congenital malformation adversely affecting life expectancy or neurodevelopment, severe IVH before randomization, and a priori palliative care. The rhEPO versus placebo comparison was not the main focus of this analysis,^31^ but since we included preterm neonates allocated to both the treatment and control arms, we evaluated a potential effect of treatment on PVS to preclude confounding by rhEPO administration (see ‘Statistical analysis’). For the present study, we used data from neonates born at the University Hospital, Zürich, and scanned at one study site, the University Children’s Hospital Zurich. Additionally, we included 44 healthy term-born neonates who were recruited as control subjects for a different study^32^ at the postnatal ward of the University Hospital Zurich from January 2011 until September 2012 and scanned at the University Children’s Hospital in Zurich. Inclusion criteria were birth >36 weeks of gestation and normal postnatal adaptation. Neonates with genetic diagnoses were excluded. Written informed consent was obtained from the parents of all eligible infants. Both studies were conducted in accordance with the Declaration of Helsinki and were approved by the ethics committee of the University Children’s Hospital Zurich, the ethics committee of the canton of Zurich, and the Swiss Agency for Therapeutic Products (Swissmedic). Neonates whose T2 MRI images had low image quality were excluded from analyses (Fig. 1). Study methods and results are reported following the Strengthening the Reporting of Observational Studies in Epidemiology Statement for cross-sectional studies.^33^

### Image acquisition and analysis

T2-weighted fast spin echo MRI data were acquired at term or term-equivalent age, with a 3T GE HD.xt scanner, using an 8-channel head coil, with TE/TR = 109/5700 ms, field of view = 25.6 cm, acquisition matrix = 256 × 256, reconstruction matrix = 512 × 512 and slice thickness = 2 mm. Axial T2-weighted images were visually inspected, and PVS counts were estimated visually in the BG and centrum semiovale (CSO) using a validated scoring system.^34^ Counts were determined by a radiologist with 7 years of experience in evaluating clinical MRI images (A.G.G.), and a subset of 10% of the images was re-evaluated by a neuroscientist with prior experience in rating PVS on T2-weighted MRI images (L.M.).^35^ Pre-defined slices consisted of the first slice above the anterior commissure for the BG and the first slice above the lateral ventricles for the CSO unless motion artefacts were present in that particular slice. Counts were converted into grades according to Potter *et al.*^34^ For all statistical analyses, we used the counts of PVSs. The PVS grades were employed solely for descriptive purposes to characterize the sample.

### White- and grey-matter scoring

White- and grey-matter abnormalities in very preterm-born neonates were assessed by two experienced investigators using the scoring system by Woodward *et al.*^36^ A detailed description of the scoring procedure can be found in Leuchter *et al.*^37^ Neonates were classified as having white matter abnormality if they had a score >6 or grey-matter abnormality if they had a score >5.^36^

### Developmental outcome

Developmental outcome in the preterm group was evaluated with the mental development index and psychomotor developmental index of the Bayley Scales of Infant Development II^38^ at ∼2 years of age. Developmental outcome in the control group was evaluated with the cognitive, language, and motor composite scores of the Bayley Scales of Infant Development III at ∼1 year of age.^39^ Developmental delay was defined using a cut-off of <70 for the Bayley II and <85 for the Bayley III.^40^

### Statistical analysis

Descriptive statistics were performed using mean/standard deviation, median/interquartile range (IQR), and *n* (%) for dichotomous variables, respectively. Groupwise differences in PVS counts were evaluated using Mann–Whitney tests. We assessed the associations of PVS counts with postmenstrual age (PMA) at MRI and preterm birth using regression analyses. In more detail, we calculated negative binomial regression models with the predictors PMA, group, sex, parental socioeconomic status (SES), and head circumference at birth as an approximate measure for head size. SES was assessed using a validated 12-point score based on maternal education and paternal occupation.^41^ We opted for negative binomial regression analyses because overdispersion was present in Poisson regression models. The predictors were selected either based on their known effects on PVSs (PMA, preterm birth,^14^ and head size^42^) or otherwise based on theoretical and biological plausibility. For all regression models, we analysed residuals to check model assumptions. Since the groups were slightly unbalanced regarding SES and PMA, we used post hoc propensity score matching to validate our results using a sample that was weighted on these variables. Propensity scores were estimated using logistic regression, and matching was performed using the full matching method. A standardized mean difference (SMD) of 0.2 was defined as cut-off for successful matching. To explore whether maturation affected PVS differentially within the preterm and control groups, we used negative binomial regression models, including a PMA by group interaction. To evaluate whether PVSs at term predicts developmental outcome (developmental delay versus no delay), we used logistic regression controlling for sex, gestational age (GA), SES and age at follow-up. We additionally compared PVSs between individuals with or without developmental delay using Mann–Whitney tests. The relationship between PVSs and white and grey matter injuries was assessed using Kendall’s rank correlation coefficient. To evaluate possible confounds from the administration of rhEPO in some of the neonates of the preterm group, we compared PVS counts between neonates receiving rhEPO and neonates receiving placebo using Mann–Whitney tests. To evaluate possible confounds by maternal chorioamnionitis, we also compared PVSs between neonates with and without maternal chorioamnionitis using Mann–Whitney tests. To address multiple testing, we applied the Bonferroni correction, dividing the traditional significance level of *α* = 0.05 by the number of tests for our primary variables of interest (*n* = 10 tests, assuming independence for the tests of preterm birth, maturation, grey matter injury, white matter injury and developmental outcome for BG and CSO PVS each), resulting in an adjusted significance level of *α* = 0.005. We assessed the inter-rater reproducibility of PVS counts using weighted Cohen’s kappa coefficients. All statistical analyses were performed with RStudio, R version 4.1.2.

## Results

### Study sample

A total of 133 neonates were evaluated, of which 89 were very preterm, and 44 were term-born neonates. Five neonates from the preterm group and one from the control group had insufficient image quality. They were excluded from all analyses, leaving a final sample size of 127 neonates, of which 84 were very preterm-born neonates (median GA 29.4, range 26–31.9 weeks) and 43 were term-born neonates (median GA 39.6, range 37.7–42 weeks) for the PVS assessments (Fig. 1). Baseline characteristics of the study sample are shown in Table 1. The control group had significantly higher parental SES (SES preterm: median = 8, IQR 8–10 versus control: median = 12, IQR 10–12; *P* < 0.001) and underwent MRI on average 1.7 weeks later than the preterm group (mean PMA at MRI 42.8 ± 1.89 weeks versus 41.0 ± 1.94 weeks for the preterm group; *P* < 0.001).

**Table 1:** Baseline characteristics

	Control (*n* = 43)	Preterm (*n* = 84)	Overall (*n* = 127)
Sex, *n* (%)			
Female	23 (53.5)	33 (39.3)	56 (44.1)
Male	20 (46.5)	51 (60.7)	71 (55.9)
Gestational age (weeks)			
Mean (SD)	39.6 (1.17)	29.2 (1.64)	32.7 (5.15)
Median (IQR)	39.6 (38.6–40.6)	29.4 (28.0–30.7)	30.7 (28.7–38.6)
Postmenstrual age (weeks)			
Mean (SD)	42.8 (1.89)	41.0 (1.94)	41.6 (2.09)
Median (IQR)	42.4 (41.6–43.8)	41.2 (39.7–42.7)	41.7 (40.4–43.0)
Chronological age (weeks)			
Mean (SD)	3.19 (1.45)	11.8 (2.47)	8.91 (4.65)
Median (IQR)	3.00 (2.29–3.79)	12.3 (10.1–13.6)	9.99 (3.79–12.9)
Birth weight (g)			
Mean (SD)	3410 (386)	1190 (318)	1920 (1100)
Median (IQR)	3390 (3060–3710)	1160 (948–1420)	1410 (1030–3060)
Missing, *n* (%)	2 (4.7)	0 (0)	2 (1.6)
Head circumference at birth (cm)			
Mean (SD)	35.1 (1.14)	26.8 (2.11)	29.6 (4.32)
Median (IQR)	35.0 (34.5–36.0)	27.0 (25.5–28.5)	28.5 (26.4–34.1)
Missing, *n* (%)	2 (4.7)	1 (1.2)	3 (2.4)
Apgar 5 score			
Mean (SD)	8.85 (0.366)	7.31 (1.80)	7.61 (1.74)
Median (IQR)	9.00 (9.00–9.00)	8.00 (6.00–9.00)	8.00 (7.00–9.00)
Missing, *n* (%)	23 (53.5%)	0 (0%)	23 (18.1%)
Umbilical arterial pH			
Mean (SD)	7.27 (0.0682)	7.45 (0.438)	7.41 (0.404)
Median (IQR)	7.29 (7.22–7.32)	7.33 (7.30–7.38)	7.33 (7.29–7.37)
Missing, *n* (%)	25 (58.1)	0 (0)	25 (19.7)
Maternal age			
Mean (SD)	33.0 (4.17)	33.1 (5.45)	33.1 (5.05)
Median (IQR)	34.0 (31.0–35.0)	34.0 (29.0–36.3)	34.0 (30.0–36.0)
Missing, *n* (%)	2 (4.7)	0 (0)	2 (1.6)
Parental socioeconomic status			
Mean (SD)	10.9 (1.53)	8.59 (2.18)	9.29 (2.26)
Median (IQR)	12.0 (10.0–12.0)	8.00 (8.00–10.0)	9.00 (8.00–11.8)
Missing, *n* (%)	7 (16.3)	2 (2.4)	9 (7.1)
Chorioamnionitis, *n* (%)			
Absent	0 (0)	64 (76.2)	64 (50.4)
Present	0 (0)	20 (23.8)	20 (15.7)
Missing	43 (100)	0 (0)	43 (33.9)

Table showing the mean (standard deviation), median (interquartile range) as well as missing data for preterm versus control group, respectively. For dichotomous variables, the absolute and relative frequencies are shown. SD, standard deviation.

### Perivascular space readout

**Figure 2:**
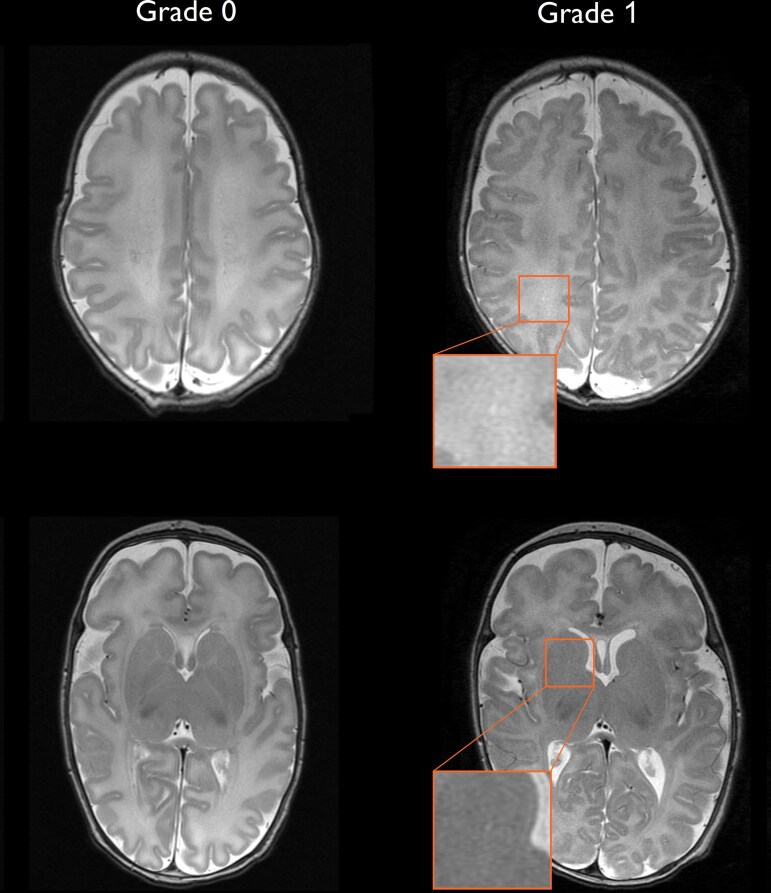
**Representative axial images illustrating Grade 0 and Grade 1 perivascular spaces in the BG and CSO.** Axial T2-weighted images from two representative neonates: one classified as Grade 0 (no visible PVS) and one as Grade 1 (presence of visible PVS). In the Grade 1 image, example PVS are magnified and marked with arrows to illustrate typical appearance and location. Image planes correspond to the pre-defined axial slices used for rating. BG, basal ganglia; CSO, centrum semiovale; PVS, perivascular space.

**Figure 3:**
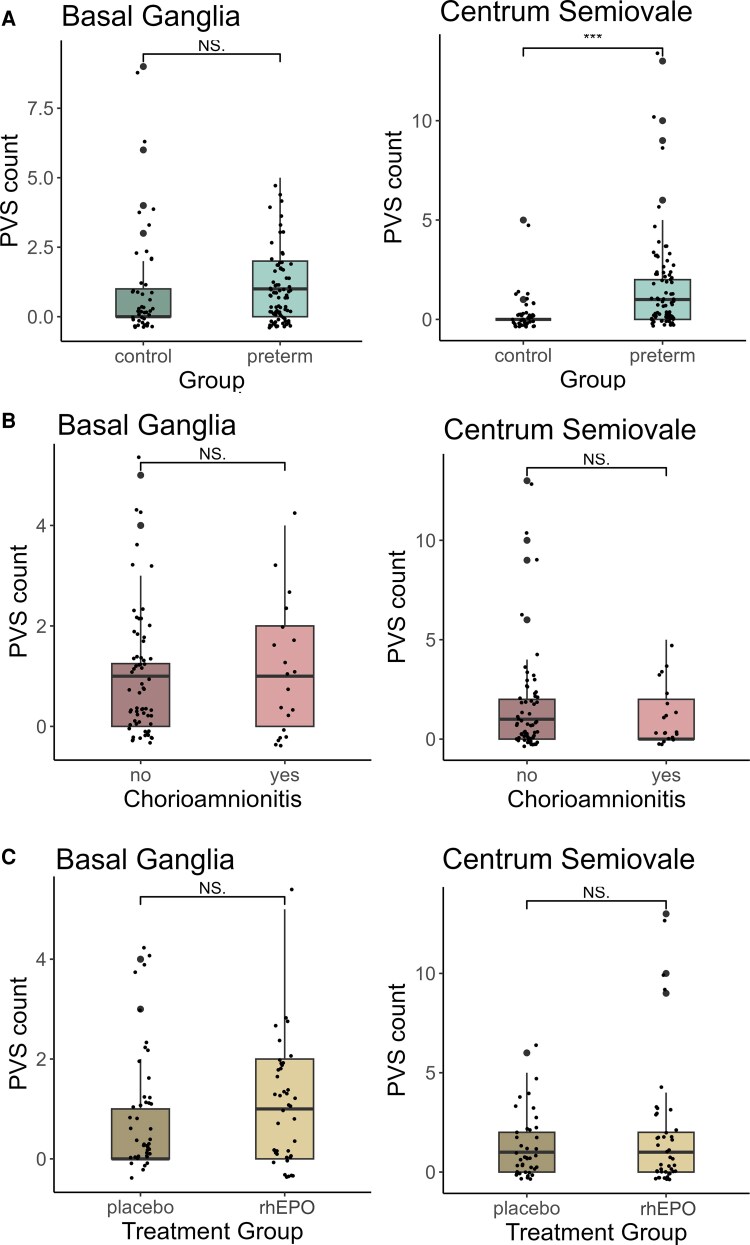
**PVS counts in the BG and CSO according to preterm birth, chorioamnionitis and treatment with erythropoietin.** Counts were determined on a pre-defined axial slice in the BG and CSO, respectively. (**A**) PVS counts in the BG did not differ between very preterm (*n* = 84) and term-born neonates (*n* = 43), whereas PVS counts in the CSO were significantly higher in preterm-born neonates (median = 1, IQR 0–2), compared with control subjects (median = 0, IQR 0–0; Mann–Whitney test; *P* < 0.001). (**B** and **C**) In preterm-born neonates, PVS counts did not differ between neonates with (*n* = 20) and without (*n* = 64) maternal chorioamnionitis nor between neonates allocated to erythropoietin treatment (*n* = 41) or placebo (*n* = 43). Data points represent PVS counts from individual neonates. BG, basal ganglia; CSO, centrum semiovale; PVS, perivascular space.

The results of the PVS readout are shown in Table 2. Apart from one subject in the preterm group, all neonates in both groups showed either Grade 0 (none) or Grade 1 (mild-grade) PVS (Fig. 2). PVS counts in the BG did not differ between groups. In contrast, PVS counts in the CSO were significantly higher in very preterm-born neonates (median = 1, IQR 0–2) compared with control infants (median = 0, IQR 0–0; *P* < 0.001; Fig. 3A). Of the 84 very preterm neonates in the present study, 41 received rhEPO and 43 received placebo in the original clinical trial. We did not find any significant difference in PVS counts between preterm neonates that received rhEPO compared with those that received placebo nor between neonates with and without maternal chorioamnionitis (Fig. 3B and C). The inter-rater reproducibility of PVS counts was *k* = 0.83, 95% confidence interval (CI) 0.67–0.99 for BG, and *k* = 0.82, CI 0.69–0.94 for CSO.

**Table 2:** Perivascular space readout

	Control (*n* = 43)	Preterm (*n* = 84)	Overall (*n* = 127)
BG PVS (count)			
Median (min, max)	0 (0, 9.00)	1.00 (0, 5.00)	0 (0, 9.00)
CSO PVS (count)			
Median (min, max)	0 (0, 5.00)	1.00 (0, 13.0)	0 (0, 13.0)
BG PVS (grade), *n* (%)			
None	26 (60.5)	39 (46.4)	65 (51.2)
Mild grade	17 (39.5)	45 (53.6)	62 (48.8)
CSO PVS (grade), *n* (%)			
None	35 (81.4)	40 (47.6)	75 (59.1)
Mild grade	8 (18.6)	43 (51.2)	51 (40.2)
Moderate	0 (0)	1 (1.2)	1 (0.8)

Table showing the median including minimum and maximum values for PVS counts in the preterm versus control group. For the PVS grades, the absolute and relative frequencies are shown. Note that PVS counts are graded in the following way: 0 = none, 1–10 = mild grade, 11–20 = moderate, 21–40 = frequent and >40 = severe.^34^ BG, basal ganglia; CSO, centrum semiovale; PVS, perivascular space.

### Maturation and preterm birth

**Figure 4:**
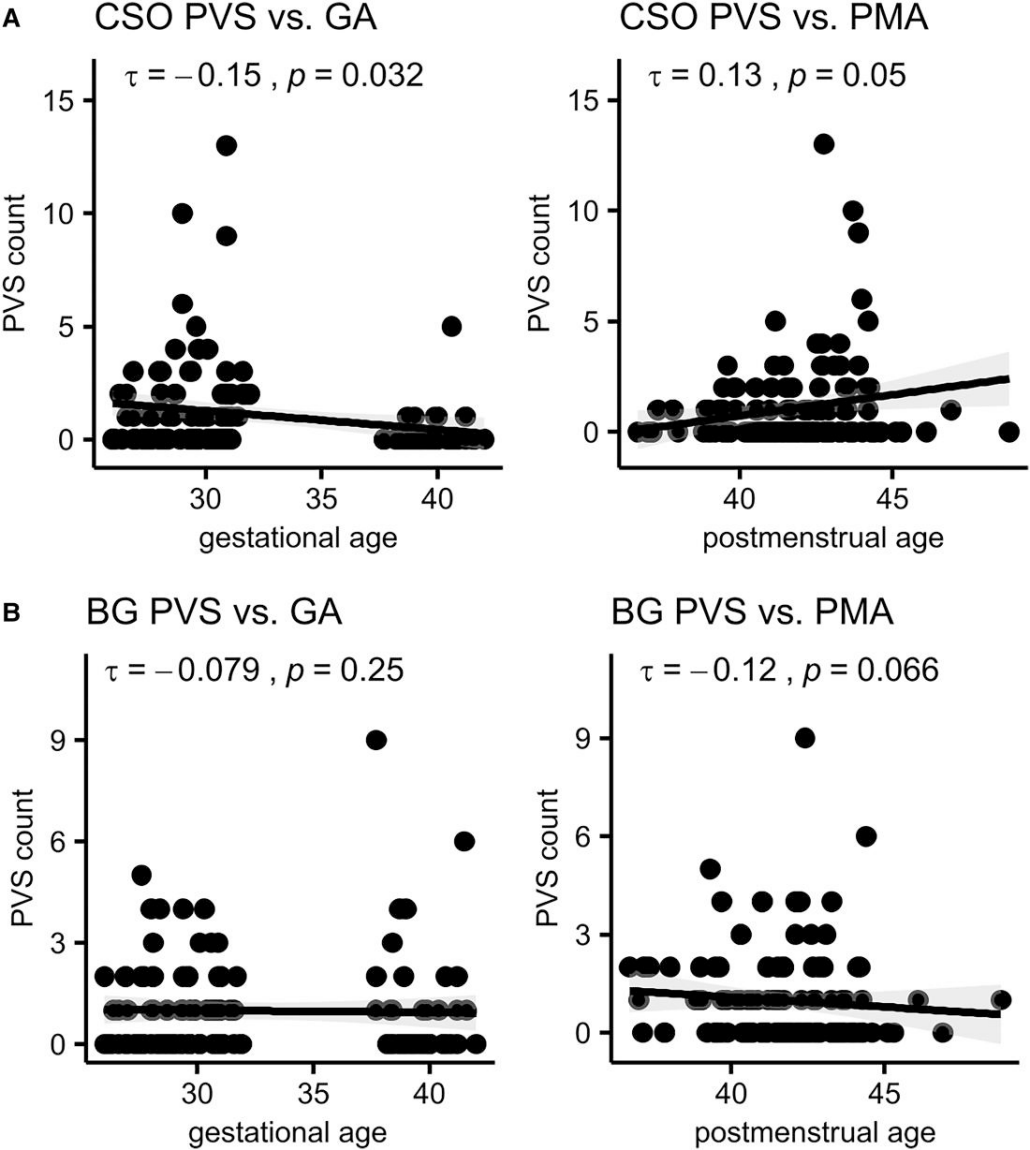
**Scatter plots for perivascular spaces versus maturation and GA.** (**A**) CSO PVS and (**B**) BG PVS. Kendall’s correlations are labelled with τ and corresponding *P*-value. Data points represent PVS counts from individual neonates (*n* = 127). BG, basal ganglia; CSO, centrum semiovale; GA, gestational age; PMA, postmenstrual age; PVS, perivascular space.

CSO PVSs were not correlated with PMA but showed an inverse relationship with GA (Fig. 4A). BG PVSs were not associated with PMA or GA but displayed a trend towards a decrease with PMA (Fig. 4B). Negative binomial regression models demonstrated that CSO PVSs increased significantly with PMA and preterm birth, independently of each other, sex, SES and head size (Table 3). Head circumference at birth additionally explained part of the variance in CSO PVSs. When including a PMA by group interaction, the interaction term was not significant, demonstrating that CSO PVSs increased similarly with maturation in both groups (*P* = 0.40). In both models, BG PVSs were not associated with preterm birth (*P* = 0.28 and *P* = 0.53) or PMA (*P* = 0.35 and *P* = 0.98). Propensity score matching resulted in a weighted sample with SMD for SES before matching: 1.23 versus after matching: −0.12 and SMD for PMA before matching: 0.9 versus after matching: −0.06 while retaining all observations (Supplementary Fig. 1). Repeating the negative binomial regression model on the weighted sample did not affect the results significantly but revealed a significant effect of sex on BG PVSs (*b* = −0.53, *P* = 0.021; Supplementary Table 1).

**Table 3:** Results of negative binomial regression models for PVS in the CSO and BG

	CSO PVS			BG PVS		
Predictors	IRR	95% CI	*P*-value	IRR	95% CI	*P*-value
(Intercept)	0.00	0.00–0.00	**<0.001**	0.94	0.00–1440.02	0.987
Preterm	44.87	10.39–212.98	**<0.001**	2.05	0.53–8.13	0.278
PMA at MRI	1.44	1.23–1.70	**<0.001**	0.94	0.82–1.07	0.347
Male	0.91	0.51–1.65	0.763	0.81	0.46–1.40	0.432
Parental SES	1.04	0.91–1.20	0.533	1.02	0.89–1.16	0.793
Head circumference	1.22	1.05–1.42	**0.011**	1.07	0.93–1.24	0.309
Observations	116			116		
*R* ^2^	0.525			0.031		

Group, postmenstrual age at MRI (weeks), sex, parental socioeconomic status and head circumference at birth (cm) were entered as predictors. Significant *P*-values are shown in bold. BG, basal ganglia; CSO, centrum semiovale; IRR, incidence rate ratio; PMA, postmenstrual age; PVS, perivascular spaces; SES, socioeconomic status.

### Brain injury and developmental outcome

Assessments of white- and grey-matter abnormalities are shown in Table 4 and assessments of developmental outcomes are shown in Table 5. PVS counts were not correlated with WMI scores (*P* = 0.92 for BG and *P* = 0.60 for CSO) nor with GMI scores (*P* = 0.75 for BG and *P* = 0.84 for CSO). Neonates who showed developmental delay at follow-up did not show a difference in PVS counts compared with neonates who showed normal development or mild developmental delay at follow-up (*P* = 0.31 for BG, *P* = 0.24 for CSO; Supplementary Fig. 2). Logistic regression models showed a significant effect of CSO PVS on developmental outcome at the 0.05 level (odds ratio = 1.27, CI 0.99–1.63; *P* = 0.047) after adjusting for GA, sex, SES and age at follow-up; however, this result did not survive multiplicity correction. BG PVSs did not affect developmental outcome after adjusting for confounders (*P* = 0.80). More details on the relationship between brain injury and developmental outcome in preterm-born neonates are shown in Supplementary Fig. 3.

**Table 4:** White- and grey-matter abnormality scores in preterm-born neonates

	Overall (*n* = 83)
Total WMI score, median (min, max)	6 (5, 11)
None	54 (64.3%)
Mild	25 (29.8%)
Moderate	3 (3.6%)
Severe	1 (1.2%)
Subscores of the WMI score, median (min, max)	
White matter signal abnormality	1 (1, 3)
Periventricular white matter loss	1 (1, 2)
Cystic abnormalities	1 (1, 3)
Ventricular dilation	2 (1, 3)
Thinning of the corpus callosum	1 (1, 3)
Total GMI score, median (min, max)	4 (3, 7)
Abnormal	1 (1.2%)
Normal	82 (97.6%)
Subscores of the GMI score, median (min, max)	
Cortical abnormality	1 (1, 3)
Gyral maturation	1 (1, 3)
Subarachnoidal space	2 (1, 3)

Table showing the median value for white- and grey-matter abnormality scores including minimum and maximum values as well as absolute and relative frequencies for the categories of the total score. Note that according to Woodward *et al*.,^36^ white matter abnormality is categorized as none (score of 5–6), mild (score of 7–9), moderate (score of 10–12) or severe (score of 13–15), whereas grey matter abnormality is categorized as normal (score of 3–5) or abnormal (score of 6–9).

**Table 5:** Developmental outcomes in term-born and preterm-born neonates

	Control (*n* = 43)	Preterm (*n* = 84)
Age at follow-up (months)		
Median (min, max)	12.7 (10.6, 23.0)	23.0 (17.3, 31.3)
Missing	8 (18.6%)	1 (1.2%)
Bayley II—MDI		
Developmental delay		6 (7.1%)
Normal development		75 (89.3%)
Missing		3 (3.6%)
Bayley II—PDI		
Developmental delay		8 (9.5%)
Normal development		73 (86.9%)
Missing		3 (3.6%)
Bayley III: cognitive		
Normal development	36 (83.7%)	
Missing	7 (16.3%)	
Bayley III: language		
Developmental delay	3 (7.0%)	
Normal development	33 (76.7%)	
Missing	7 (16.3%)	
Bayley III: motor		
Developmental delay	1 (2.3%)	
Normal development	35 (81.4%)	
Missing	7 (16.3%)	

Absolute and relative frequencies of neonates with and without developmental delay or with missing data, respectively, for Bayley subscales. For the preterm group, outcome was assessed with the Bayley Scales of Infant Development II at ∼1 year of age, while for the control group, outcome was assessed with the Bayley Scales of Infant Development III at ∼2 years of age. For the Bayley II, a score <70 was used as cut-off for developmental delay, and for the Bayley III, a score of <85 was used as cut-off. MDI, mental development index; PDI, psychomotor developmental index.

## Discussion

In this study, we investigated the relationship between PVSs, maturation, preterm birth, brain injury and developmental outcomes in very preterm and at-term neonates. Our results suggest that CSO PVSs increase with maturation and that preterm birth affects CSO PVS counts.

### Perivascular space readout

Only one other study investigated PVSs in preterm and term-born neonates, demonstrating that MRI-visible PVSs are present in neonates but seem to exceed Grade 1 (<10 PVS) only rarely.^14^ Correspondingly, all neonates in our sample were either graded 0 (0 PVSs) or 1 (<10 PVSs), apart from a single preterm neonate with Grade 2 CSO PVSs. According to the grading system by Wardlaw *et al.*,^43^ Grade 1 corresponds to ‘mild-grade’ PVSs; however, since this system was developed for adults, it is uncertain whether the observed grades possess clinical relevance in neonatal patients, such as preterms. In the present study, very preterm-born neonates showed no difference in BG PVS counts but had more PVSs in CSO compared with controls. Interestingly, Kim *et al.*^14^ reported no difference in CSO PVSs but lower grade BG PVSs in preterm-born neonates. The difference between our results and those reported previously may be due to the overall lower GA in preterm neonates in our sample or due to the high variability and differential sensitivity of the manual scoring method and the MRI protocol. For example, 40% of neonates in our sample had Grade 1 CSO PVS versus 6% of neonates in Kim *et al.*^14^

### Relationship of PVS with brain maturation

When assessing the relationship of PVSs with maturation (PMA), we found that CSO PVSs but not BG PVSs were positively associated with PMA both before and after controlling for group, sex, SES and head size, suggesting that CSO PVSs increase with maturation in both very preterm and term-born neonates. Kim *et al*.^14^ found a negative association of PMA with the BG PVS volume fraction but did not assess the relationship between BG PVS counts and PMA. Although we did not fully replicate this result, our data show a trend towards a negative association of BG PVS counts with maturation. Therefore, since BG PVS counts are known to decrease with age in children and adults before increasing again in older age,^12^ they may decrease only slightly in neonates, which would fit with the negative trend in our sample. The group by PMA interaction was neither significant nor improved the model fit, demonstrating that CSO PVSs increased at similar rates in both groups. Thus, preterm birth does not speed up the maturation-related increase in PVSs but contributes to the PVS burden independently of maturation, potentially via secondary mechanisms (see the following section). The observed increase in white matter PVSs with PMA is in line with the results of large cross-sectional studies, reporting the strongest increases in white matter PVSs early in life.^12,13^ Interestingly, the white matter PVS burden in later life has been suggested to be inversely related to the childhood PVS burden, such that white matter regions with low PVS volume fraction early in life show the greatest increase in adulthood.^12^ Owing to this critical link between PVS physiology in early life and ageing, future longitudinal studies should explore whether preterm birth leads to region-specific changes in white matter PVS morphology and whether this affects their trajectory in later life. Taken together, our results suggest that CSO PVSs in neonates increase with maturation, independently of preterm birth.

### Relationship of PVS with preterm birth

In the present study, we identified significantly higher CSO PVSs in very preterm-born neonates compared with controls, and results of our regression analyses confirmed that the effect of group on CSO PVSs was also significant after controlling for PMA, sex, SES and head size. A possible reason for the observed effect of preterm birth could be that CSF secretion and glymphatic activity increase in response to higher metabolic demands owing to an accelerated grey and white matter development after preterm birth.^44,45^ As outlined before, the neurofluid system is in an immature state in preterm-born neonates and, therefore, increases in CSF secretion might result in the accumulation of interstitial fluid (ISF) within the PVS. Another potential mechanism linking preterm birth to enlarged PVSs could be diffuse white matter injury, but our results and those of Kim *et al.*^14^ suggest that PVSs are not associated with white matter injury. On the other hand, white matter PVSs are thought to colocalize with axon fibre tracts, and when visible on MRI, these dilated ISF spaces have been suggested to represent mild local axonal atrophy rather than glymphatic dysfunction in adults.^46^ Correspondingly, the higher numbers of white matter PVSs we observed in our preterm cohort could point to disrupted myelination in the brains of very preterm-born individuals. Such findings have been reported previously (see, e.g. Back^47^ and Dibble *et al*.^48^) and would offer an explanation as to why CSO PVSs—and not BG PVSs—are associated with preterm birth in our cohort. Taken together, the potential mechanisms underlying any effect of preterm birth on CSO PVSs, including neurofluid drainage dysfunction, impaired myelination or increased metabolic demands, remain unclear and warrant further investigation.

### Brain injury and developmental outcomes are not linked to PVS in neonates

In the present study, both grey- and white-matter injury scores showed no association with BG PVSs or CSO PVSs. This result is in line with Kim *et al.*,^14^ who compared PVSs according to the presence or absence of punctate white matter lesions and found no difference between the groups.^49^ Oligodendrocytes, the myelinating cells of the central nervous system, are particularly vulnerable to oxidative stress resulting from preterm birth, and consequent white matter injury is accompanied by inflammation, diffuse gliosis and altered extracellular matrix composition.^47^ In an adult brain, these processes would most likely obstruct neurofluid drainage pathways, leading to perivascular oedema.^27^ However, it is possible that neurofluid dynamics differ substantially in neonates. For example, a higher brain water content with a gradual shift of water from extracellular to intracellular compartments,^50^ differences in brain barrier mechanisms,^51^ venous pressure^52,53^ or higher expression levels of Aquaporin-4 channels in neonates^54,55^ could allow for a more rapid CSF–ISF exchange, supporting the brain during critical phases of growth and development. Accordingly, although we identified a significant effect of CSO PVSs on developmental outcome (*P* = 0.047), this effect did not survive multiplicity correction. Therefore, our data suggest that PVSs are not associated with early developmental outcomes in preterm or term-born neonates. However, given that preterm birth is associated with increased risks for long-term neurological and neuropsychiatric conditions, including attention-deficit/hyperactivity disorder (ADHD), autism spectrum disorder and cognitive or emotional difficulties,^28^ early developmental assessments may not fully capture the long-term impact of neonatal brain alterations. Enlarged PVSs or increased PVS counts have been reported in infants with high familial risk for autism^56^ and in older children with ADHD^57^ or developmental delay.^58^ Thus, increased PVSs may represent an early marker of altered neurodevelopmental trajectories that only become clinically apparent later in life, even if they do not relate to early developmental delays. One possible mechanism involves inflammation during critical periods of development which may alter brain barrier mechanisms and predispose individuals to neurologic or neuropsychiatric diseases, in which the integrity of the blood–brain barrier is compromised.^59,60^ Future longitudinal studies should investigate PVSs in newborns in relation to neurodevelopmental outcomes as well as blood–brain barrier and neurofluid clearance functions across the lifespan.

### Limitations

Our study had several limitations. First, there was a time difference of 1.7 weeks between the median PMA at MRI in the preterm and control groups. Considering the rapid developmental changes in the postnatal period, it is possible that the preterm group would show even higher numbers of CSO PVSs if they were imaged at a median PMA of ∼43 weeks, like the control group. Secondly, the groups were unbalanced regarding parental SES. To account for these biases, we used propensity score matching to create a weighted sample on which we repeated the regression analysis. Furthermore, developmental outcomes were assessed at different time points and with different versions of the Bayley scales, but we accounted for this by dichotomizing into neonates with and without developmental delay and used logistic regression to check whether PVSs predicted delay while controlling for sex, GA, SES and age at follow-up. The use of data from a study of rhEPO is also a limitation, even though no significant effects of rhEPO were observed on neurodevelopmental outcome at 2 years.^31^ Similarly, no significant association between rhEPO treatment and PVSs was observed in our analysis, but the possibility that rhEPO may influence vascular development or white matter integrity^37,49^—and thus PVSs—cannot be fully excluded and should be considered when interpreting these findings. Lastly, the single-slice visual assessment of PVSs may be considered a limitation; however, we assessed the reliability of our readout by re-rating a subset of 10% of our images and achieved very good inter-rater agreement. Future work could benefit from automated methods, which may offer additional insights into PVS volume, diameter, and solidity, reduce potential bias and enhance comparability across studies.

## Conclusion

These results demonstrate an association of CSO PVSs with preterm birth and an increase of CSO PVSs with maturation. Longitudinal studies may shed further light on the potential effects of preterm birth on the brain’s neurofluid clearance system development.

## Supplementary Material

fcaf244_Supplementary_Data

## Data Availability

The data underlying this article can be shared on reasonable request with the corresponding author, with permission from the relevant ethics committee.
