# Supplementary material for: Prematurity is associated with white matter T2 MRI visible perivascular spaces in very preterm-born neonates
Source: Brain Commun. 2025 Jun 18;7(3):fcaf244. doi: 10.1093/braincomms/fcaf244 (PMC12203554; doi:10.1093/braincomms/fcaf244)
Supplement: fcaf244_Supplementary_Data [file fcaf244_supplementary_data.docx]

**Prematurity is Associated with White Matter T2 MRI Visible Perivascular Spaces in Very Preterm Born Neonates**

Lena Meinhold^1^, Antonio G. Gennari^1^, Giancarlo Natalucci², Beatrice Latal^3^, Flavia Wehrle^3^, Jean-Claude Fauchere², Cornelia Hagmann^4^, Ruth O'Gorman Tuura^1^

**Authors Affiliations**

^1^Center for MR Research, University Children’s Hospital, Zurich, Switzerland

²Department of Neonatology, University Hospital Zurich, Switzerland

^3^Child Development Centre, University Children’s Hospital, Zurich, Switzerland

^4^Department of Neonatology, University Children’s Hospital Zurich, Switzerland

Correspondence to: Lena Meinhold

University Children`s Hospital Zurich, Centre for MR Research, Lenggstrasse 30, 8008 Zürich, Switzerland

Lena.Meinhold@kispi.uzh.ch

**Supplementary Material**

**Supplementary Table 1. Negative binomial regression models showing the relationship between PVS, maturation and preterm birth using a weighted sample.**

|  | **CSO PVS** | | | **BG PVS** | | |
| --- | --- | --- | --- | --- | --- | --- |
| *Predictors* | *IRR* | *95% CI* | *p* | *IRR* | *95% CI* | *p* |
| (Intercept) | 0.00 | 0.00 – 0.00 | **<0.001** | 0.46 | 0.00 – 350.05 | 0.816 |
| grouppreterm | 24.68 | 5.85 – 111.33 | **<0.001** | 2.25 | 0.71 – 7.17 | 0.158 |
| postmenstrual age [weeks] | 1.55 | 1.33 – 1.83 | **<0.001** | 0.93 | 0.82 – 1.05 | 0.254 |
| sexmale | 0.84 | 0.48 – 1.48 | 0.542 | 0.59 | 0.37 – 0.92 | **0.021** |
| parental SES | 1.10 | 0.96 – 1.26 | 0.162 | 1.03 | 0.92 – 1.16 | 0.543 |
| head circumference [cm] | 1.22 | 1.06 – 1.42 | **0.008** | 1.11 | 0.99 – 1.26 | 0.082 |
| Observations | 116 | | | 116 | | |
| R^2^ Nagelkerke | 0.597 | | | 0.099 | | |

Propensity score matching was used to create a balanced sample on the variables PMA and SES, using the full matching method. A standardized mean difference (SMD) of 0.2 was defined as cut-off for successful matching (see figure below). (IRR: incidence rate ratio, BG: basal ganglia, CSO: centrum semiovale, SES: socioeconomic status).

**Supplementary Fig. 1. Covariate balance before and after propensity score weighting.** Standardized mean differences (SMDs) are shown for covariates postmenstrual age at scan and parental socioeconomic status (SES). The dashed line indicates the SMD threshold of 0.2 for acceptable balance.


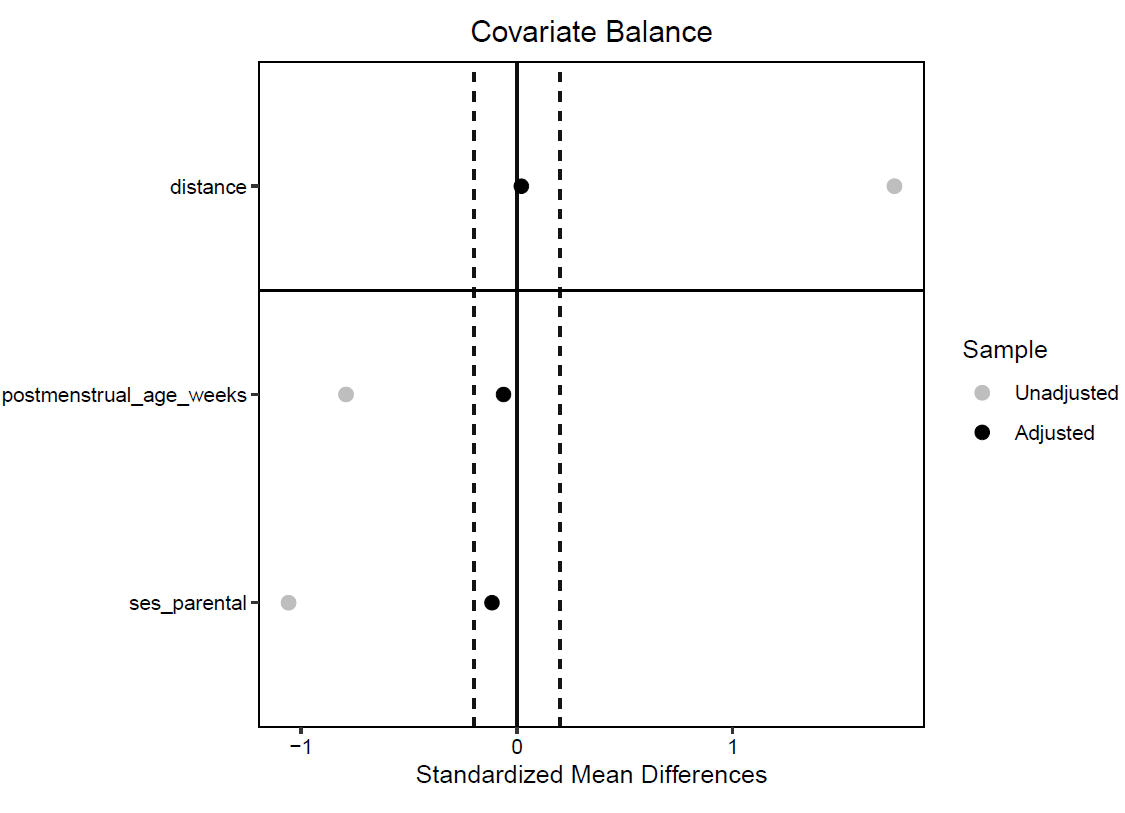


**Supplementary Fig. 2. Centrum semiovale and basal ganglia PVS counts for preterm neonates with normal development versus developmental delay.** Neonates with developmental delay (N=16) did not show more PVS counts compared to neonates with normal development (N=100; Mann–Whitney test, *p*=0.31 for BG, *p*=0.24 for CSO), Data points represent PVS counts from individual neonates (N=116). (PVS: perivascular space, CSO: centrum semiovale, BG: basal ganglia).

**
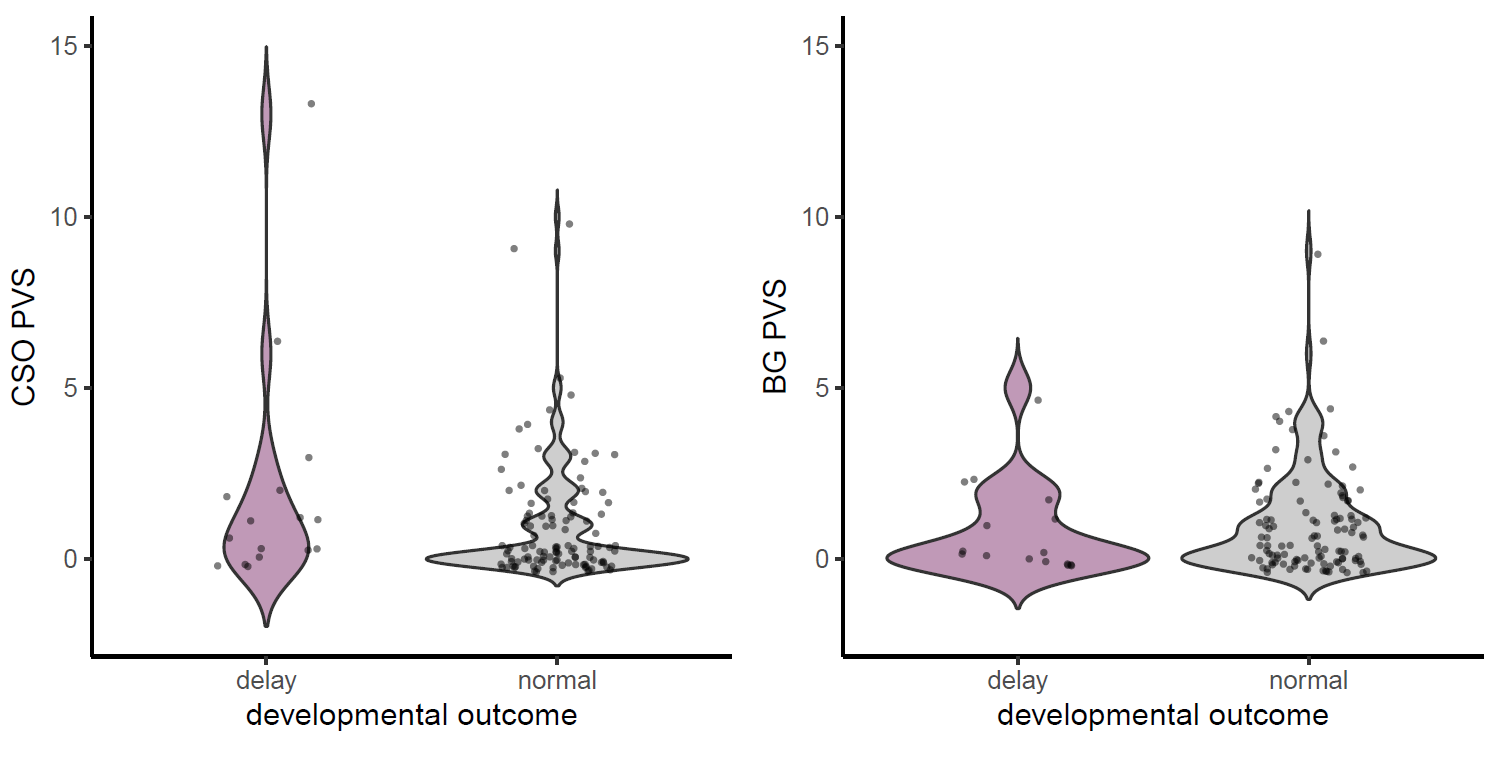
**

**Supplementary Fig. 3. Scatter plots for white and gray matter injury versus Bayley II Scale in very preterm born neonates.** (**A**) White matter injury and (**B**) gray matter injury. Kendall`s correlations are labelled with 𝜏 and corresponding p value. Data points represent brain injury scores and outcomes from individual neonates (N=80). (WMI: white matter injury, GMI: gray matter injury, MDI: mental development index, PDI: psychomotor development index)


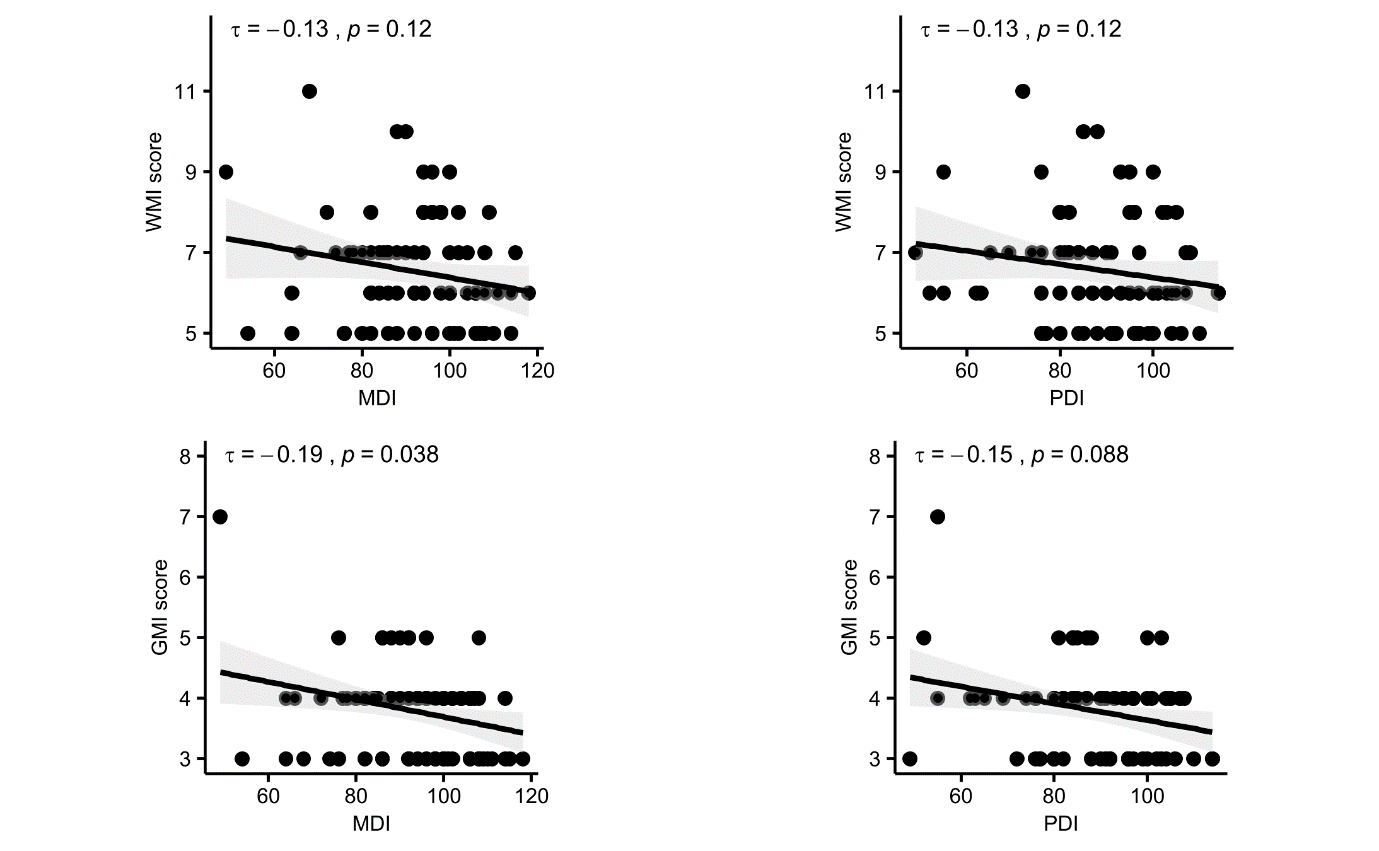


**A**

**B**
